# Beliefs and strategies about urinary incontinence: a possible moderation role between symptoms and sexual function, and quality of life

**DOI:** 10.3389/fpsyg.2023.1252471

**Published:** 2023-11-30

**Authors:** Marta Porto, João Marôco, Teresa Mascarenhas, Filipa Pimenta

**Affiliations:** ^1^William James Center for Research, Ispa – Instituto Universitário, Lisbon, Portugal; ^2^FLU Pedagogy, Nord University, Bodø, Norway; ^3^Department of Obstetrics and Gynecology, CHSJ-EPE/Faculty of Medicine, University of Porto, Porto, Portugal

**Keywords:** beliefs, female urinary incontinence, functional urology, moderation, quality of life, sexual function, strategies

## Abstract

**Background:**

Urinary Incontinence (UI) has numerous repercussions in women’s lives, and it is underreported/underdiagnosed.

**Objective:**

The present study aimed to understand: (1) the differences between women with and without urine loss regarding Quality of Life (QoL) and Sexual Function (SF); (2) the possible moderation role of UI-related beliefs and strategies on the relationship between UI-symptom severity and SF and QoL, in women with UI.

**Methods:**

Cross-sectional Design. Participants: Primary aim: Overall, 2,578 women aged 40–65 (*M*_age_ = 49.94, *DP*_age_ = 6.76) were collected online. Secondary aim: 1,538 women who self-reported having urine loss occasionally/frequently (*M*_age_ = 50.19, *DP*_age_ = 6.58). All data analyses were done with IBM SPSS Statistics and R statistical system 4.0 through RStudio. Statistical Path analysis was performed with the lavaan package to study the hypothetical association and moderating effects between the variables.

**Results:**

Primary aim: women without UI had a better SF [*t*(2576) = 3.13, *p* = 0.002; 95% C.I., 0.18 to 0.80] and QoL [*t*(2576) = 7.71, *p* < 0.001; 95% C.I., 3.14 to 5.28] than their counterparts with UI. Secondary aim: UI-related coping strategies attenuated the impact of UI-symptom severity on SF(*β* = −0.07; *p* = 0.041); the more dysfunctional the UI-related beliefs were, the poorer QoL was (*β* = −0.06; *p* = 0.031); the more frequent the UI-related hiding/defensive strategies were, the poorer QoL was (*β* = −0.26; *p* < 0.001).

**Discussion:**

Limitations: online data collection, which thwarted the clarification of participants, if needed; absence of a UI medical diagnosis (only self-reported measures were used). Strengths and practical implications: (i) the crucial role of UI-related beliefs and strategies in the QoL of women with UI; (ii) the impact that UI-concealing/defensive strategies have in attenuating the impact of UI-symptom severity on SF, which might be perceived as a short-term benefit and hence contribute to maintaining the UI condition and constitute a barrier to help-seeking, (iii) impact of UI-symptom severity on QoL and SF (including a comparison group entailing women without UI, which is scarcely used); and (iv) the use of gold-standard and psychometrically robust instruments.

**Conclusion:**

Changing dysfunctional UI-related beliefs and strategies in clinical settings may improve the QoL; UI-concealing strategies may reinforce themselves by immediate effects on SF, but are not functional in the long term.

## 1 Introduction

According to the Abrams et al. (2009), Urinary Incontinence (UI) is defined as the complaint of any involuntary loss of urine (Haylen et al., 2010; D’Ancona et al., 2019; Frawley et al., 2021). Although UI is not a life-threatening disease, the loss of urine involves numerous physical (e.g., urine odor, frequent urinary infections), psychological (e.g., stigma, shame, dysfunctional coping), sexual (e.g., loss of urine during intercourse, sexual dysfunction, decreased libido), sociocultural (e.g., social isolation, limitations in activities of daily living, precocious institutionalisation of the elderly), professional (e.g., absenteeism, lower productivity), and economic/financial repercussions (e.g., increased expenditure on underwear and pads and diapers) (Shaw et al., 2019) (Subak et al., 2006; Abrams et al., 2015; Caruso et al., 2017). These consequences lead to poorer Quality of Life (QoL) in women, highlighting that the psychosocial effect can be more devastating than the physical consequences. In this way, these numerous repercussions may lead to the implementation of lifestyle changes and coping strategies, which can be (dys) functional and are based on an individual’s illness representations (Minassian et al., 2012; Waetjen et al., 2018).

The Commonsense Model of Self-Regulation (CSM) (Leventhal et al., 2016) is a dynamic conceptual framework that allows understanding cognition (i.e., individual UI beliefs) and coping strategies, explains health-related behaviors and can be used to guide health promotion and health education interventions by identifying the gaps in knowledge that present the most adverse effects on help-seeking behaviors. According to this regulatory model, individuals simultaneously create a cognitive and emotional representation of the illness/health threat (Fernandez-Duque and Schwartz, 2016; Leventhal et al., 2016). Cognitive representation has five dimensions: (1) identity (i.e., UI label and the individual’s ideas about the somatic representation UI), (2) timeline (i.e., expected duration of UI), (3) causality (i.e., beliefs about the perceived cause of UI), (4) consequences (i.e., imagined *consequences* or anticipated repercussions in terms of personal experiences, economic hardship, or the emotional upheaval of the UI) and (5) curability/controllability (i.e., UI responsiveness to self and/or professional intervention) (Hagger and Orbell, 2003; Leventhal et al., 2016; Fan et al., 2017). These representations are processed through three stages: (1) firstly, a representation of the illness or health threat (i.e., UI representations) appears; (2) secondly, the adoption of behaviors to cope with the illness takes place (i.e., coping strategies); and (3) lastly, it occurs the evaluation of the efficacy of these behaviors (i.e., appraisal of coping strategies) (Hagger and Orbell, 2003).

Moreover, identifying the types of beliefs about UI could explain individual differences in help-seeking behavior and related outcomes (such as coping strategies adopted and compliance with medical regimens) (Minassian et al., 2012; Bascur-Castillo et al., 2019). Literature documents that only 25% of affected women seek care, and less than half receive treatment (Minassian et al., 2012). Those who seek medical help will tend to consult a primary healthcare clinician and rarely will be referred to a clinician with training in incontinence management (Lucas et al., 2012). Depending on their beliefs, women may tend to self-manage UI, perpetuating symptoms (Toye and Barker, 2020; Mahfouz et al., 2023). Since women’s dysfunctional beliefs toward UI (e.g., the belief that UI is a normal ageing characteristic) are considered one of the barriers to seeking health care, understanding, and changing them is crucial (Bush et al., 2001; Cooper et al., 2015).

On experiencing the first symptoms of UI, women may face other factors that also impact healthcare-seeking: (1) embarrassment about leakage, (2) low confidence regarding health professionals (e.g., patients’ self-perceived *symptoms devaluation* by *medical professionals*), (3) lack of knowledge about treatment options and; (4) adaptation of their daily routine to UI symptoms (Lasserre et al., 2009). The latter may require the use of UI dysfunctional coping strategies, including defensive ones (e.g., limiting social and physical activities; avoiding sexual intercourse) or/and strategies intended to conceal urine loss (e.g., wearing pads and/ or diapers) (Diokno et al., 2004). These maladaptive strategies might also impair Quality of Life (QoL). This is congruent with many studies: women with UI present an impairment of their Quality of Life and Sexual Function (SF) (e.g., sexual embarrassment, vaginal dryness, loss of urine during sex, decreased libido) (e.g., Abrams et al., 2015; Felippe et al., 2017). However, most studies have explored Sexual Function and Quality of Life without comparing their counterparts with no urine loss. For example, Bilgic and Kizilkaya Beji (2020), who studied the impact of UI types on the aforementioned variables, concluded that one of their study’s limitations was the lack of a control group without UI. Likewise, Fatton et al. (2014) found that the presence of UI was significantly associated with poor SF, but only focused on women with UI. Moreover, little is known about the role of UI-related beliefs, as well as UI-related coping strategies.

In this way, more studies are needed to address this empirical knowledge gap and better understand: (1) the differences between women with and without urine loss regarding QoL and SF (primary aim); (2) the possible moderation role of UI-related beliefs and strategies on the relationship between UI symptoms’ severity and SF and QoL, in women with UI (secondary aim).

## 2 Materials and methods

### 2.1 Design

The current study follows a cross-sectional design.

### 2.2 Participants

#### 2.2.1 Primary aim

Overall, 2,648 women, aged 40 to 65 (*M*_age_ = 49.94, *DP*_age_ = 6.76), were inquired online (community non-probabilistic sample). Inclusion criteria were (1) age (40–65 years), (2) sex (women), and (3) internet access. Exclusion criteria were (1) Pregnancy or delivery in the past 6 months. Due to this exclusion criteria, 40 women were excluded, resulting in a total of 2,578 women.

#### 2.2.2 Secondary aim

One thousand five hundred and thirty-eight women who self-reported having urine loss occasionally/frequently (*M*_age_ = 50.19, *DP*_age_ = 6.58), from the abovementioned sample, were selected. Inclusion criteria were (1) age (40–65 years), (2) sex (women), (3) women who initially responded affirmatively in the research protocol to the question regarding involuntary urine leaks when coughing or experiencing the urge to urinate occasionally or frequently, and (4) internet access. Exclusion criteria included (1) pregnancy or until 6 months post-partum, (2) previous UI-related surgery, (3) abdomen, gynecological and breast cancer, (4) neurological disease and (5) pelvic organ prolapse. Fifty-six women were excluded since they had had surgery for UI.

### 2.3 Measures

#### 2.3.1 Sociodemographic and clinical characterization

Self-reported questionnaires collected sociodemographic characteristics (age, nationality, level of education, professional status, sexual-affective relationship, number of biological children), as well as clinical (number of vaginal deliveries, number of caesarean deliveries, perineal laceration, physical and mental illness diagnosis, menopausal status, BMI Status), lifestyle characteristics (coffee intake, hot and cold beverages intake, high impact physical activity) and UI-related information (medical help for UI, treatment for UI, prescribed UI medication, and Pelvic Floor Muscle Training).

UI types were assessed using the QUID questionnaire (Bradley et al., 2010), specifically designed for diagnosing UI in women. The cut-off points for diagnosis were stress scores ≥4 and urge scores ≥6. Mixed UI type indicated the presence of both QUID diagnoses.

The menopausal status was defined according to the Stages of Reproductive Aging Workshop‘s criteria (STRAW) (Soules et al., 2001). Pre-menopausal women self-reported as not having any changes in their menstrual cycle. Peri-menopausal participants self-reported a variable cycle length (a difference of more than 7 days than usual) or had skipped two or more cycles and experienced an amenorrhea interval of more than 60 days. Post-menopausal women self-reported having experienced at least 12 months of amenorrhea (Tables 1, 2).

**Table 1:** Sociodemographic characteristics of participants.

Characteristics	Women with UI (*n* = 1,538)		Women without UI (*n* = 1,040)		Total (*N* = 2,578)	
	*n*	%	*n*	%	*n*	%
Age range						
40–44	353	23.3	298	28.3	662	24.4
45–49	392	25.9	232	23.3	638	24.9
50–54	368	24.3	224	21.0	592	23.1
55–59	266	15.9	181	17.4	422	16.4
60–65	159	10.5	105	10.0	264	10.3
Nationality						
Portuguese	1,482	96.3	1,010	97.2	2,492	96.6
Other	56	3.7	30	2.8	86	3.4
Level of Education						
4^th^ grade	6	0.4	4	0.4	10	0.4
9^th^ grade	118	7.8	60	7.0	192	7.5
12^th^ grade	447	29.5	305	28.9	763	29.3
Bachelor’s degree	573	37.9	422	40.0	995	38.8
Postgraduate degree	358	22.0	225	21.3	558	21.7
Doctorate	36	2.4	24	2.3	600	2.3
Professional status						
Active	1,245	80.6	810	78.2	2055	79.6
Inactive	293	19.4	230	21.8	523	20.4
Sexual-affective relationship						
Yes	1,222	79.1	859	81.5	2056	80.1
No	316	20.9	181	18.5	522	19.9
Number of biological children						
0	152	10.0	148	14	300	11.7
1	462	28.9	338	32.1	786	30.2
2	695	45.9	452	44.2	1,161	45.2
3	185	12.2	82	7.8	267	10.4
> 4	44	2.9	20	1.9	64	2.5

UI, Urinary incontinence.

**Table 2:** Clinical characteristics and lifestyle habits of participants.

Characteristics	Women with UI (*n* = 1,538)		Women without UI (*n* = 1,040)		Total (*N* = 2,578)	
	*n*	%	*n*	%	*N*	%
Number of vaginal deliveries						
0	425	28.1	460	43.6	885	34.3
1	442	27.6	248	24.9	690	26.8
2	509	33.6	268	25.4	777	30.1
3	131	8.7	54	5.1	185	7.2
4	31	2.0	10	0.9	41	1.6
Number of caesarean deliveries						
0	1,062	70.2	636	60.3	1,698	65.9
1	325	19.8	242	23.0	567	22.0
2	121	8.0	135	14.1	256	9.9
3	28	1.9	26	2.5	54	2.1
4	2	0.1	61	0.1	63	2.4
Perineal laceration						
Yes	851	54.6	403	38.2	1,254	48.6
No	687	45.4	637	61.8	1,324	51.4
Physical illness diagnosis						
Yes	545	34.4	276	26.2	821	31.8
No	993	65.6	764	73.8	1757	68.2
Asthma	45	2.9	32	3.1	77	3.0
Hypothyroidism	51	3.3	35	3.4	86	3.3
Diabetes	35	2.2	22	2.1	57	2.2
Hypertension	72	4.7	50	4.8	122	4.7
Mental illness diagnosis						
Yes	248	14.7	116	11.0	364	14.1
No	1,290	85.3	924	89.0	2,214	85.9
Depressive disorder	133	8.8	57	5.4	190	7.4
Anxiety disorder	96	4.7	46	4.4	142	5.5
Obsessive disorder	4	0.3	2	0.2	6	0.2
Psychotic disorder	5	0.3	8	0.8	13	0.5
Menopausal status						
Premenopause	339	22.4	262	24.9	601	23.31
Perimenopause	574	36.3	322	31.9	896	34.8
Postmenopause	625	41.3	456	43.3	1,081	41.9
Medical help to manage menopausal symptoms						
Yes	664	43.9	441	43.2	1,105	42.9
No	874	56.1	599	56.8	1,473	57.1
Coffee intake						
Yes	1,263	83.5	854	82.4	2,117	82.1
No	275	16.5	186	17.6	461	17.9
Hot/cold beverages intake						
Yes	1,286	85.0	868	83.7	2,154	83.6
No	252	15.0	172	16.3	424	16.4
High impact physical activity						
Yes	207	13.7	183	17.4	390	15.13
No	1,331	86.3	857	82.6	2,188	84.87
BMI status						
Underweight	32	2.1	5	1.8	37	1.4
Healthy weight	651	41.4	491	46.6	1,142	44.3
Overweight	552	36.5	350	33.2	902	35.0
Obesity level I	226	14.9	158	15.0	384	14.9
Obesity level II	60	4.0	32	3.0	92	3.6
Obesity level III	17	1.1	4	0.4	21	0.81
Medical help for UI						
Yes	620	41.0				
No	918	59.0				
Treatment for UI						
Yes	448	448				
No	1,090	1,090				
Prescribed UI medication						
Yes	70	4.6				
No	1,468	95.4				
PFMT						
Yes	369	24				
No	1,169	76				

UI, Urinary incontinence; PFMT, Pelvic Floor Muscle Training.

#### 2.3.2 Exogenous variables

##### 2.3.2.1 King’s Health Questionnaire – 5 severity-related items

An unidimensional 5-item index (“Do you wear pads to keep dry?”; “Frequency: Going to the toilet very often”; Stress Incontinence: Urine leakage during physical activity (e.g., coughing, running”), “Nocturnal Enuresis: Wetting the bed at night”; Intercourse Incontinence: Urine leakage with sexual intercourse”) driven from King’s Health Questionnaire (Kelleher et al., 1997; Viana et al., 2015) (a self-administered questionnaire that assesses both the presence of UI symptoms and their impact on Quality of Life), was used to measure UI symptom severity. The Likert-type response scale ranged from 1 – “*Not at all*” to 4 – “*A Lot*.” Higher score levels indicate higher symptom severity (total score ranged between 5 and 25). Total Scores under the middle point (i.e., 12,5) indicated Mild to Moderate UI-symptom Severity, while total scores above the middle point indicated moderate to severe UI-symptom severity. Regarding psychometric sensibility, there were no severe violations of normality, given that all items presented adequate absolute values of skewness and kurtosis (sk < 3; ku < 7). All items presented adequate standardized factor weights (0.51 < *λ* < 0.74) and adequate individual reliability (*R*^2^ > 0.25). Goodness-of-fit indices indicated a good fit of the final factor structure (CFI = 0.961; TLI = 0.921; RMSEA = 0.091; SRMR = 0.044), indicating good factorial validity. The convergent validity evidence based on the internal structure was good (AVE = 0.53). The data gathered with FSFI-6 showed good reliability (*α* = 0.83; *ω* = 0.51).

#### 2.3.3 Endogenous variables

##### 2.3.3.1 Female Sexual Function Index

The Portuguese version of the Female Sexual Function Index (FSFI-6) (Isidori et al., 2010; Pimenta et al., 2017) measures women’s sexual functioning. It is a 6-item short version of the 19-item Sexual Functioning Index. Each item refers to a dimension related to sexual functioning: desire (e.g., “In the last four weeks, how would you rate your level (degree) of sexual desire or interest?”), arousal (e.g., “In the last four weeks how would you rate your level (degree) of sexual arousal during intercourse or vaginal penetration?”), lubrication (e.g., “Over the past four weeks, how often have you felt lubricated (noticed more vaginal discharge) during sexual activity or vaginal penetration?”), orgasm (e.g., “Over the last four four weeks, how often have you had an orgasm when you have had sexual stimulation or penetration?”), satisfaction (e.g., “Over the past four weeks, how satisfied have you been with your sexual activity?”), and pain (e.g., “Over the past four weeks, how often have you felt vaginal discomfort or pain with penetration?”). The items are scored on a 5-point Likert scale. The total score varies between 2 and 30 points (4 questions including 0), with higher values indicating better sexual functioning. A cutoff point less than or equal to 19 points identifies individuals with low sexual functioning. Regarding psychometric sensibility, there were no severe violations of normality, given that all items presented adequate absolute values of skewness and kurtosis (sk < 3; ku < 7). All items presented adequate standardized factor weights (0.58 < *λ* < 0.93), and adequate individual reliability (*R*^2^ > 0.25). Goodness-of-fit indices indicated a good fit of the final factor structure (CFI = 0.992; TLI = 0.987; RMSEA = 0.127; SRMR = 0.038). In This way, FSFI-6 presented good factorial validity. The convergent validity evidence based on the internal structure was good (AVE = 0.67). The data gathered with FSFI-6 showed good reliability (*α* = 0.92; *ω* = 0.914).

##### 2.3.3.2 WHO-QOL

The Portuguese version of WHO Quality-of-Life-Bref (Skevington et al., 2004; Canavarro et al., 2010), assesses QoL generically and multi-dimensionally. The WHOQOL-Bref consists of 26 items, assessed on a 5-point Likert scale from 1 (e.g., “*Not at all*”) to 5 (e.g., “*Completely*”), comprising four quality of life domains: physical (e.g., “To what extent do you need medical care to carry out your daily life?”), psychological (“Are you able to accept your physical appearance?”), social relationships (e.g., “How satisfied are you with your personal relationships?”), and environment (e.g., “Until How satisfied are you with the transport you use?”). Regarding psychometric sensibility, there were no severe violations of normality, given that all items presented adequate absolute values of skewness and kurtosis (sk < 3; ku < 7). All items presented adequate standardized factor weights (0.503 < *λ* < 0.920) and adequate individual reliability (*R*^2^ > 0.25). Goodness-of-fit indices indicated a good adjustment of the final factor structure, with the correlation between the residuals of items 1 and 2 Goodness-of-fit indices indicated an acceptable fit of the factor structure (CFI = 0.919; TLI = 0.907; RMSEA = 0.101; SRMR = 0.065), indicating factorial validity. The four dimensions abovementioned presented adequate average variance extracted values, indicating convergent validity. Discriminant validity was verified between the subscales. Regarding reliability, the four subscales presented adequate reliability (Physical: *α* = 0.94; *ω* = 0.92; Psychological: *α* = 0.86; *ω* = 0.80; Social Relationships: *α* = 0.85; *ω* = 0.80; Environment: *α* = 0.867; *ω* = 0.80).

#### 2.3.4 Moderation variables

##### 2.3.4.1 Illness Perception Questionnaire-Brief

The Portuguese version of the Brief Illness Perception Questionnaire (IPQ-Brief) (Broadbent et al., 2006; Figueiras et al., 2012) was used to assess the cognitive representations of UI. IPQ-Brief has a unifactorial structure with eight items: five items appraising cognitive illness through (1) consequences (“How much does your illness affect your life?”), (2) timeline (“How long do you think your illness will continue?”), (3) personal control (“How much control do you feel you have over your illness?”), (4) treatment control (“How much do you think your treatment can help your illness?”), and (5) identity (“How much do you experience symptoms from your illness?”); two assessing emotional representations (“How concerned are you about your illness?” / “How much does your illness affect you emotionally? (e.g., does it make you angry, scared, upset or depressed?”); and one assessing illness comprehensibility (“How well do you feel you understand your illness?”). These eight items are rated on a response scale ranging from 0 (e.g., does not affect at all) to 10 (e.g., severely affects my life). The last item is a causal open-response item, adapted from the IPQ-R (Moss-Morris et al., 2002), which asks patients to list the three main causal factors in their illness. The total score is generated by summing up the scores for the Brief-IPQ items with a reverse scoring of items 3, 4 and 7. The total score varies between 10 and 80 and a higher total score reflects a more threatening perception of illness. Regarding psychometric sensibility, there were no severe violations of normality, given that all items presented adequate absolute values of skewness and kurtosis (sk < 3; ku < 7). All items presented good factor loadings (*λ* ≥ 0.50), with adequate individual item reliability (*R*^2^ ≥ 0.25) except for item 4 (*λ* = −0.358), item 7 (*λ* = 0.119), which presented very low loadings, and item 3 (*λ* = 0.372), which was not removed due to its phenomenological and clinical relevance. After the elimination of these two items (4 and 7), most goodness-of-fit indices were indicative of a good fit to the data (CFI =0.994; TLI = 0.990; RMSEA = 0.111; SRMR = 0.024). The convergent validity evidence based on the internal structure was good (AVE = 0.60). Regarding reliability, the data gathered with Brief IPQ showed good reliability (*α* = 0.92; *ω* = 0.86).

##### 2.3.4.2 UI-related coping strategies instrument

A bi-factorial instrument developed by our team that entails a set of UI-related maladaptive coping strategies to manage the immediate effects of UI based on the work of Diokno et al. (2004). The “hiding coping” dimension is constituted by five strategies whose purpose is concealing urine stains strategies (e.g., “Use feminine hygiene pads,” “Wear dark clothes to conceal urine stains,” “Use panty liners”), and the “defensive coping” dimension, which includes eight strategies to control urine loss (e.g., “Limit fluid intake,” “Limit social activities,” “Limit physical exercise”). A Likert scale was used from 1 - “Never” to 5 - “Always.” The total score varies between 13 and 65, and a higher score reflects a more frequent use of coping strategies. Regarding psychometric sensibility, there were no severe violations of normality, given that all items presented adequate absolute values of skewness and kurtosis (sk < 3; ku < 7). All items presented adequate standardized factor weights (0.54 < *λ* < 0.952) and adequate individual reliability (*R*^2^ > 0.25). Goodness-of-fit indices indicated a good fit of the factor structure (CFI = 0.986; TLI = 0.984; RMSEA = 0.074; SRMR = 0.058), indicating factorial validity. Both the Defensive and Hiding subscales had adequate average variance extracted values of 0.69 and 0.54, respectively, indicating convergent validity. Discriminant validity was verified between the subscales (*r*^2^ = 0.402). Regarding reliability, both subscales presented good reliability (Defensive: *α* = 0.94; *ω* = 0.92; Hiding: *α* = 0.80; *ω* = 0.83). This is the first validation study.

### 2.4 Data collection

The study data was collected online between March and October 2020 using the online survey platform Google Forms (including all the material described above). When inviting the participants online (i.e., via social media - Facebook), and disseminating a brief description of the research in several posts in groups of middle-aged/menopause-related women, the research purpose was clearly explained.

#### 2.4.1 Ethics issue

This study is part of the research project (PURI-PRO: Portuguese Urinary Incontinence Project), which aims to contribute to the promotion of a healthier life and well-being for middle-aged women (specifically, menopausal women), meeting the United Nations 2030 Agenda. Participants were given a link to an informed consent statement and the online survey questionnaire and were assured that participation was anonymous and voluntary. Informed consent was obtained from all participants. The main goal was explained, the study’s procedures and that they could stop answering whenever they wanted and that anonymity and confidentiality were ensured since no identification data was requested, and a code was attributed to each participant. Non-responses were not allowed and participants could return to verify and/or correct the answer to each survey before submission. The leading researcher’s contact number was provided for any inquiries. The present study was approved by The Ethical Committee for Research from ISPA- Instituto Universitário and followed the ethical guidelines and the standards of the Portuguese Psychologists Association (Portuguese Psychologist Association, 2011), and the American Psychological Association (American Psychological Association, 2003).

### 2.5 Data analysis

All data analyses were done with the IBM SPSS Statistics, version 27, and with R 4.0 (R Core Team, 2020), through RStudio (R Core Team, 2020) (v. 26).

#### 2.5.1 Primary aim

Confirmatory Factor Analyses (CFA) were performed to assess psychometric properties in the present sample for all the instruments used. CFA used Robust Maximum Likelihood estimation as implemented in the lavaan package (v.0.6-14) for the R statistical system (Rosseel, 2012). After attesting for construct validity evidence for each construct, a multigroup analysis was performed to show the equivalence of the proposed model across women with and without UI, to verify if the differences in scale scores are caused by differences in levels of the underlying construct, not other causes (e.g., items’ interpretation). Configural, metric, scalar, and strict invariance were tested using nested models with increasing loadings, intercepts, and error variance constraints. Values of |ΔCFI| < 0.01 (Cheung and Rensvold, 2002), and |ΔRMSEA| ≤ 0.02 (Rutkowski and Svetina, 2014) between consecutive nested models indicated that the model was invariant in women with and without urine loss. After confirming invariance and the assumptions of the dependent variable’s normality and homogeneity of variance, Student’s *t*-tests were run on the data with a 95% confidence interval (CI) for the mean difference between women with and without urine loss, regarding QoL and SF.

#### 2.5.2 Secondary aim

Pearsons’ Correlations were used to verify the possible association between UI-symptom severity and (1) UI-related beliefs and (2) UI-related strategies to proceed with the planned analysis (i.e., without association between these variables, a moderation model would not be plausible). The Pearson correlations were considered high if >0.50, low below <0.20, and moderate if in between (Marôco, 2021).

After the association of these variables was verified, the data normality was assessed. No severe deviations from the normal distribution that would be recommended against parametric testing were considered for absolute values of Kurtosis (Ku) smaller than seven and Skewness (Sk) smaller than three (Kline, 2016). Additionally, the mean interpolation method imputed missing values for variables whose frequency was lower than 5% of the sample. Multicollinearity between the independent variables was also evaluated with the variance inflation factor (VIF) (Marôco, 2021). A value below five indicates the absence of multicollinearity. Finally, the existence of outliers was evaluated through the Mahalanobis distance (Marôco, 2021).

Path analysis was performed with the lavaan package for the R statistical system (R Core Team, 2020) to study the hypothetical association and moderating effects between the variables under study (i.e., evaluate whether UI-related beliefs and strategies would moderate the effects of UI symptom severity on SF and QoL). Moreover, the model goodness of fit was evaluated using the Comparative Fit Index (CFI), the Root Mean Square Error of Approximation (RMSEA), the Tucker-Lewis Index (TLI) and the Standardized Root Mean Square Residual (SMRM) (Cheung and Rensvold, 2002). CFI and TLI above 0.9, as well as SRMR and RMSEA below 0.08, were indicative of good model fit (Byrne, 2010; Marôco, 2021).

To better understand the relationship between strategies and sexual function an additional analysis was made, namely a multigroup analysis comparing women who are sexually active and women who are not sexually active regarding the impact of strategies on sexual function. Additionally, we adjusted the moderation model to the three different types of IU (i.e., Stress UI, Urgency UI and Mixed UI) to explore whether these groups have differences regarding QoL.

A 5% level of significance was used for the decision-making regarding the statistical significance of the results.

## 3 Results

### 3.1 Clinical characterization of the UI sub-sample

Regarding UI types, 558 (36.2%) women presented symptoms congruent with a UI stress pattern, 334 (21.7%) women with an urgency pattern, and 243 (16.1%) with a mixed pattern. The other 26% of women did not meet the criteria for diagnosis since their total score was under the cut-point. Regarding UI severity, 1,200 (78%) presented mild to moderate UI-symptom severity, and 338 (22%) presented moderate to severe UI.

Concerning sexual function, 405 (27%) women did not have sexual intercourse during the last 4 weeks; 269 (17.7%) women reported that UI affected the relationship with their partner; 309 (20.1%) women reported that UI affected their sexual life; and 427(28.2%) women lost urine during sexual intercourse.

### 3.2 Primary aim

All instruments presented evidence of good psychometric properties (reliability, sensitivity, and construct validity – factorial, convergent and discriminant validity) in the present study.

The model showed scalar/strong invariance (|ΔCFI ≤0.01|, |ΔRMSEA ≤0.02|) for both QoL and SF for women with and without urine loss. It was found that the total score of FSFI in women without involuntary loss of urine (*M* = 20.23, *SD* = 3.78) was significantly higher than in women with involuntary loss of urine (*M* = 19.74, *SD* = 4.01), [*t*(2576) = 3.13, *p* = 0.002; 95% C.I., 0.18–0.80]: women without UI had a better SF, compared with their counterparts with UI.

Moreover, it was also found that the total score of WHO-QOL in women without UI (*M* = 98,43, *SD* = 12.56) was significantly higher than in women with involuntary loss of urine (*M* = 94.22, *SD* = 14.37), [*t*(2576) = 7.71, *p* < 0.001; 95% C.I., 3.14–5.28]: women without UI had a better QoL, compared with their counterparts with UI.

### 3.3 Secondary aim

Pearson correlations were calculated to investigate whether UI symptom severity was associated with UI-related beliefs and strategies. Significant associations among UI and UI-related beliefs [*r*(1513) = 0.714, *p* < 0.001] and strategies were found [*r*(1513) = 0.705, *p* < 0.001].

**Figure 1:**
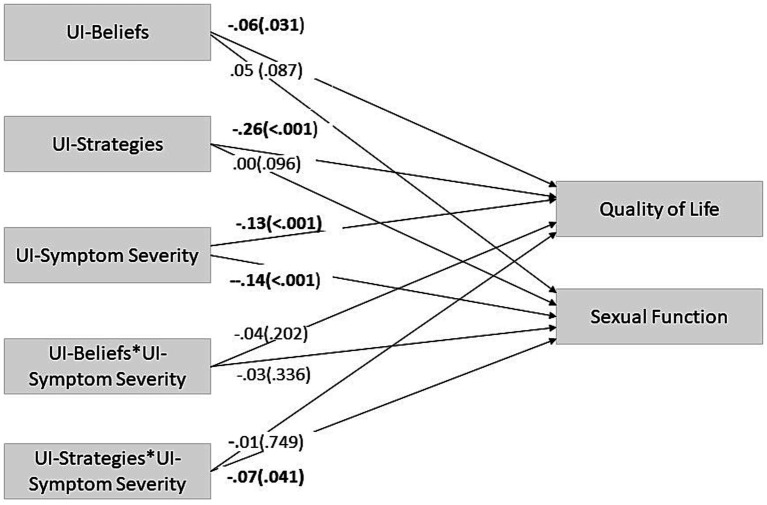
Path analysis model – moderation model. This path analysis model shows UI-related beliefs and strategies as possible moderators of the relation between Urinary Incontinence and Quality of Life and Sexual function. The coefficients presented are standardized linear regression coefficients. Significant trajectories’ values are in bold. UI, Urinary Incontinence. * = x. ^a^standardized β (value of *p*).

The obtained model of path analysis was saturated. UI-related coping strategies significantly moderated the effects of UI symptom severity on Sexual Function (SF) (i.e., the presence of UI-related strategies to manage the immediate effects of UI attenuated the impact of UI-symptom severity on SF). Also, UI-related beliefs did not moderate the relation between UI-symptom severity and SF. Regarding Quality of Life (Qol), neither UI-related strategies nor UI-related beliefs moderated the effects of UI symptom severity on QoL (Figure 1).

Finally, the direct main effects of UI symptom severity were significant on both QoL and SF: women with more severe UI symptoms reported poorer QoL/SF. Regarding UI-related beliefs and strategies, their main effects were only significant on QoL: on the one hand, the more dysfunctional the UI-related beliefs were, the poorer QoL was; on the other, the more frequently the dysfunctional UI-related strategies were used, the poorer QoL was (Figure 1).

### 3.4 Additional analysis

To better understand the relationship between strategies and sexual function an additional analysis was made, namely a multigroup analysis comparing women who are sexually active and women who are not sexually active regarding the impact of strategies on sexual function. The model was not invariant [Δ*X*^2^_λ_ (13) = 38.901; *p* < 0.001], which means that there are differences between sexually and non-sexually active women, regarding the impact of UI-related strategies on Sexual Function. In sexually inactive women, UI-related strategies did not significantly impact Sexual Function (*β*_defensive_ = −0.085; *p* = 0.096; *β*_hiding_ = −0.034, *p* = 0.513). However, in sexually active women, the impact of UI-related strategies on Sexual Function was significant (*β*_defensive_ = −0.117; *p* < 0.001; *β*_hiding_ = −0.066, *p* = 0.046) (i.e., the more dysfunctional the UI-related beliefs were, the poorer Sexual Function was).

Additionally, we adjusted the moderation model to the three different types of IU to explore whether these groups have differences regarding QoL. The direct effect of UI-symptom severity was significant on QoL in women with Mixed UI (*β* = −0.174, *p* = 0.043). In this way, the more severe UI symptom, the worse QoL was). The direct effect of UI-symptom severity was not significant either for women with Stress UI (*β* = −0.051, *p* = 0.390), nor for women with Urgency UI (*β* = −0.139, *p* = 0.191).

## 4 Discussion

The present study aimed to explore how UI symptoms impact Quality of Life (QoL) and Sexual Function (SF), also tackling the moderating role of UI-related beliefs and strategies in the relationship between UI-symptom severity, and both QoL and SF.

### 4.1 Direct effects

Regarding the direct effect of UI-symptom severity on QoL and SF, our results support previous research in the field of UI, which links symptom severity and QoL (e.g., Lasserre et al., 2009; Abrams et al., 2015) and SF (e.g., Fatton et al., 2014; Bilgic and Kizilkaya Beji, 2020) (although in the absence of a comparison group with women without UI, in previous studies). Several studies report women with UI symptomatology as having poorer QoL and SF^17^, lower libido, lubrification, and satisfaction, as also shown by our data. Our findings also highlight the role of UI-related beliefs and strategies in the QoL of women with UI. In this sense, the more frequent UI-related strategies are (in this study, characterized by hiding and defensive behaviors), the more negative the impact on QoL is. This is consistent, especially considering three QoL domains and the detrimental effect that some of the UI-management strategies used by these women might have on these domains: (1) physical (UI-related strategy example: “reduce physical activity”), (2) psychological (UI-related strategy example: “staying at home to avoid uncomfortable situations”; and (3) social relationships (UI-related strategy example: “reduce social gatherings”). For example, to enhance QoL, women with SUI might follow a Pelvic Floor Muscle Training protocol to reduce UI symptoms, as Pires et al. (2020) concluded.

Contrary to our predictions, the direct impact of UI-related strategies was not observed on SF. One possible explanation may be that QoL is a broader construct, being easier to be impacted by hiding/ defensive behaviors to manage UI. Another explanation might be due to the fact that there are differences between sexually and non-sexually active women, regarding the use of UI-strategies on Sexual Function.

The direct impact of UI-related beliefs was also not observed in SF. This result might be explained by the fact that UI-related beliefs (measured in our study by IPQ-Brief) include dimensions related to symptoms, control/cure, timeline, and general consequences, and do not include dimensions related to SF, such as shame, and intimacy. Future studies should assess UI-related beliefs more closely related to sexual function/distress (e.g., fear of UI’s impact on sexual interaction).

### 4.2 Moderation

Concerning moderation, these results represent the first direct demonstration of the moderation role of UI-related strategies in the relationship between UI-symptom severity and Sexual Function, as far as the authors know. In this sense, UI-related strategies aiming to control UI symptoms (which include “reduce fluid intake,” “go to the bathroom often, even if you do not feel like it,” and “use feminine/sanitary pads”) attenuate the relationship between UI and SF. Due to the efficacy of these strategies and consequent reduction of UI symptoms during sexual intercourse, women may be more available for sexual intercourse. If, on the one hand, these control behaviors may have value in alleviating and controlling symptoms in the immediate (i.e., immediately before or during sexual intercourse), on the other hand, they also perpetuate UI symptoms in the long term since women do not seek medical help and maintain these hiding and defensive behaviors. Additionally, the moderation effect, but not the direct effect of the UI management strategies in SF, might have been shown because this sample shows only mild to moderate UI-symptom severity levels.

### 4.3 Limitations

There are three potential limitations concerning the results of this study. The first limitation is related to online data collection because it limits the opportunity to clarify questions, if they emerge during the protocol fulfillment. A second limitation is that the findings are based on individual self-reports (in the absence of a medical diagnosis), which may contribute to participant bias. A third potential limitation is that the only source of sexual activity characterization was the Female Sexual Function Index (FSFI-6). Finally, most women in the present sample presented a mild to moderate UI degree.

### 4.4 Strengths

Despite these limitations, these results suggest several theoretical and practical implications and strengths: (i) the crucial role of UI-related beliefs and strategies (both modifiable through brief interventions) in the QoL of women with UI; (ii) the impact that UI-hiding/defensive strategies have in attenuating the impact of UI-symptom severity on SF, which might be perceived as a short-term benefit and hence contribute to maintaining the UI condition and constitute a barrier to help-seeking, (iii) confirmation of the impact of UI-symptom severity on QoL and SF (including a comparison group entailing women without UI, which is scarcely used); and (iv) the use of gold-standard and psychometrically robust instruments.

### 4.5 Future research

Regarding future research, it would be helpful to extend the current findings by examining the role of UI-related beliefs and strategies among the different UI types (i.e., Stress UI, Urgency UI and Mixed UI). Future studies should also contemplate the assessment of vaginal dryness as it relates to menopausal transition and might have a confusional effect on sexual functioning in middle-aged women. It would also be useful for future research to assess both relationship and sexual satisfaction, and use specific UI-related beliefs measures. Finally, the fact that data collection was done in a pandemic period, including phases of mandatory lockdown, might have impacted Sexual Function and Quality of Life values. Future studies should address these variables in non-pandemic periods to explore if the report of the variables changes.

## 5 Conclusion

Regarding translation to practice and although the generality of the current results must be established by future research, the present study has provided clear support for the importance of assessing and changing UI-related beliefs and strategies in clinical settings (among multidisciplinary teams - including urologists and psychologists for middle-aged women) to improve the efficacy of UI healthcare interventions and providing tailored urological health promotion regimens.

## Data availability statement

The raw data supporting the conclusions of this article will be made available by the authors, without undue reservation.

## Ethics statement

The studies involving humans were approved by ISPA – Instituto Universitário Ethics Committee. The studies were conducted in accordance with the local legislation and institutional requirements. The participants provided their written informed consent to participate in this study.

## Author contributions

MP: protocol/project development, data collection, data analysis, methodology, manuscript writing, project administration. FP: project development, review and editing, supervision, validation, project administration. TM: review and editing, conceptualization of medical aspects. JM: methodology, review and editing, supervision, data analysis, psychometric validation. All authors contributed to the article and approved the submitted version.
