# Corrigendum: Beliefs and strategies about urinary incontinence: a possible moderation role between symptoms and sexual function, and quality of life

**DOI:** 10.3389/fpsyg.2024.1359674

**Published:** 2024-02-09

**Authors:** Marta Porto, João Marôco, Teresa Mascarenhas, Filipa Pimenta

**Affiliations:** ^1^William James Center for Research, Ispa – Instituto Universitário, Lisbon, Portugal; ^2^FLU Pedagogy, Nord University, Bodø, Norway; ^3^Department of Obstetrics and Gynecology, CHSJ-EPE/Faculty of Medicine, University of Porto, Porto, Portugal

**Keywords:** beliefs, female urinary incontinence, functional urology, moderation, quality of life, sexual function, strategies

In the published article, there was an error in the author list order. The corrected author list should be as follows:

Marta Porto^1*^, João Marôco^1,2^, Teresa Mascarenhas^3^ and Filipa Pimenta^1^

The affiliations have also been corrected to account for the change in author order. Additional information has also been added to make the affiliations more complete affiliations. They should be as follows:

^1^William James Center for Research, Ispa – Instituto Universitário, Lisbon, Portugal

^2^FLU Pedagogy, Nord University, Bodø, Norway

^3^Department of Obstetrics and Gynecology, CHSJ-EPE/Faculty of Medicine, University of Porto, Porto, Portugal

In addition, in the published article the Acknowledgement statement was not included. The correct Acknowledgement statement can be found below:

“Our team is thankful to Teresa Garcia Marques, who gave important input regarding the importance of having a comparison group (i.e., women without Urinary Incontinence).”

The authors apologize for these errors and state that they do not change the scientific conclusions of the article in any way. The original article has been updated.

