# Supplementary material for: Beliefs and strategies about urinary incontinence: a possible moderation role between symptoms and sexual function, and quality of life
Source: Front Psychol. 2023 Nov 30;14:1252471. doi: 10.3389/fpsyg.2023.1252471 (PMC10720902; doi:10.3389/fpsyg.2023.1252471)
Supplement: Supplementary file 1 [file Data_Sheet_1.docx]

| Please mark the frequency with which you use each of the following strategies to manage bladder leakage (0 - Never, 1- Rarely, 2- Sometimes, 3- Often, 4- Always) |
| --- |
| **Defensive** |
| Go to the toilet frequently, even when you do not have urge, just to keep the blader empty. |
| Limit intake of fluids. |
| Avoid going places where do not know location of the toilet. |
| Stay at home more to avoid uncomfortable situations. |
| Limit physical activity. |
| Learn in advance where toilets are before going to an unfamiliar place. |
| Limit social situations. |
| Limit activities, such as travel, that take you away from the toilet for a long period of time. |
| **Hiding** |
| Use adult diapers. |
| Use panty liners. |
| Use other absorbent materials (such as toilet paper, tissue, paper towels). |
| Wear dark clothing or colours that will not show stains. |
| Wear long shirts or jackets to cover stains. |
|  |
|  |
| ***Translation to Portuguese:*** |
| **Instrumento de Estratégias de Coping para Incontinência Urinária** |
| Por favor, assinale a frequência com que utilizou cada uma das estratégias indicadas para gerir perdas de urina. (Nunca, Raramente, Algumas vezes, Muitas vezes, Sempre) |
| **Estratégias defensivas** |
| Ir muitas vezes à casa de banho, mesmo sem vontade, de forma a manter a bexiga vazia. |
| Reduzir a ingestão de líquidos. |
| Evitar ir a locais onde desconhece a localização das casas de banho. |
| Ficar mais tempo em casa de forma a evitar situações desconfortáveis. |
| Limitar a atividade física. |
| Antes de se deslocar a um local desconhecido, tentar saber onde se situam as casas de banho. |
| Limitar as saídas sociais. |
| Limitar atividades, como viajar, que requerem estar longe da casa de banho durante um longo período. |
| Estratégias de ocultação |
| Usar cuecas-fralda. |
| Usar pensos femininos (higiénicos). |
| Usar outros materiais absorventes (tais como papel higiénico, lenços de papel ou toalhas de papel). |
| Usar cores ou roupas escuras que escondam as manchas. |
| Usar saias e casacos compridos para cobrir as manchas. |

Table 3 - UI-related Coping Strategies Instrument (Appendix)
